# *Ku70/80* gene expression and DNA-dependent protein kinase (DNA-PK) activity do not correlate with double-strand break (dsb) repair capacity and cellular radiosensitivity in normal human fibroblasts

**DOI:** 10.1038/sj.bjc.6690166

**Published:** 1999-03

**Authors:** U Kasten, N Plottner, J Johansen, J Overgaard, E Dikomey

**Affiliations:** Institute of Biophysics and Radiobiology, University of Hamburg, Martinistrasse 52, 20246 Hamburg, Germany; Department of Experimental Clinical Oncology, Aarhus University Hospital, Nørrebrogade 44, Aarhus C, Denmark

**Keywords:** DNA-dependent protein kinase, *Ku70/80*, double-strand break repair, radiosensitivity, normal human fibroblasts

## Abstract

The expression of the *Ku70* and *Ku80* genes as well as the activity of the DNA-dependent protein kinase (DNA-PK) were studied in 11 normal human fibroblast lines. The proteins studied are known to be part of a double-strand break (dsb) repair complex involved in non-homologous recombination, as was demonstrated for the radiosensitive rodent mutant cell lines of the complementation groups 5–7. The 11 fibroblast lines used in this study represent a typical spectrum of normal human radiosensitivity with the surviving fraction measured for a dose of 3.5 Gy, SF_3.5 Gy_, ranging from 0.03 to 0.28. These differences in cell survival were previously shown to correlate with the number of non-repaired dsbs. We found that the mRNA signal intensities of both *Ku70* and *Ku80* genes were fairly similar for the 11 cell lines investigated. In addition, the DNA-PK activity determined by the pulldown assay was fairly constant in these fibroblast lines. Despite the correlation between cell survival and dsb repair capacity, there was no correlation between dsb repair capacity and DNA-PK activity in the tested normal human fibroblast lines. Obviously, in this respect, other proteins/pathways appear to be more relevant. © 1999 Cancer Research Campaign


British Journal of Cancer (1999) 79(7/8), 1037–1041
© 1999 Cancer Research Campaign
Article no. bjoc.1998.0166

### *Ku70/80* gene expression and DNA-dependent protein kinase (DNA-PK) activity do not correlate with double-strand break (dsb) repair capacity and cellular radiosensitivity in normal human fibroblasts

**U Kasten[1], N Plottner[1], J Johansen[2], J Overgaard[2] and E Dikomey[1]**

[1]Institute of Biophysics and Radiobiology, University of Hamburg, Martinistrasse 52, 20246 Hamburg, Germany; [2]Department of Experimental Clinical Oncology, Aarhus University Hospital, Nørrebrogade 44, 8000 Aarhus C, Denmark

**Summary** The expression of the *Ku70* and *Ku80* genes as well as the activity of the DNA-dependent protein kinase (DNA-PK) were studied in 11 normal human fibroblast lines. The proteins studied are known to be part of a double-strand break (dsb) repair complex involved in non-homologous recombination, as was demonstrated for the radiosensitive rodent mutant cell lines of the complementation groups 5–7. The 11 fibroblast lines used in this study represent a typical spectrum of normal human radiosensitivity with the surviving fraction measured for a dose of 3.5 Gy, $SF_{3.5\ Gy}$, ranging from 0.03 to 0.28. These differences in cell survival were previously shown to correlate with the number of non-repaired dsbs. We found that the mRNA signal intensities of both *Ku70* and *Ku80* genes were fairly similar for the 11 cell lines investigated. In addition, the DNA-PK activity determined by the pulldown assay was fairly constant in these fibroblast lines. Despite the correlation between cell survival and dsb repair capacity, there was no correlation between dsb repair capacity and DNA-PK activity in the tested normal human fibroblast lines. Obviously, in this respect, other proteins/pathways appear to be more relevant.

**Keywords:** DNA-dependent protein kinase; *Ku70/80*; double-strand break repair; radiosensitivity; normal human fibroblasts

In radiotherapy, the maximum dose used to cure a patient from a tumour is partially limited by the occurrence of severe late normal tissue reactions. The extent of these reactions varies individually and shows a broad spectrum even after identical or comparable schemes of irradiation (Bentzen and Overgaard, 1994; Turesson et al, 1996).

There is now increasing evidence that the extent of late normal tissue reactions may depend to a considerable extent on the intrinsic cellular radiosensitivity (Geara et al, 1993; Bentzen and Overgaard, 1994; Ramsay and Birrell, 1995; Burnet et al, 1996; Johansen et al, 1996). So far, the factor(s) determining the intrinsic cellular radiosensitivity are not definitely known. For normal human fibroblasts, radiation-induced loss of cellular reproductivity is known to result from chromosomal damage (Cornforth and Bedford, 1987) and also from a permanent arrest at the $G_1$/S border (Di Leonardo et al, 1994). Both of these effects appeared to be determined by non- or misrejoined DNA double-strand breaks (dsbs). This was recently observed in our laboratory for a variety of normal human fibroblasts (Dikomey et al, 1999), where a good correlation between the cellular radiosensitivity and the number of non-repaired double-strand breaks was found.

Up to now, the best known complex involved in mammalian double-strand break repair is the DNA-dependent protein kinase (DNA-PK) complex. This complex consists of the DNA binding subunits Ku70/80 (*XRCC6/XRCC5*) and the catalytic subunit DNA-PKcs (*XRCC7*) and is known to be part of the non-homologous recombination pathway, which is considered to be the dominant dsb repair mechanism active in mammalian cells (Jackson and Jeggo, 1995). Additionally, in primate cells, this protein complex interacts with the Ku80-related protein KARP-1 (Myung et al, 1997). In rodent cells, any defect in this complex was found to result in a depressed repair capacity and an enhanced cellular radiosensitivity (Zdzienicka, 1995). However, in human cell lines (mostly human tumour cell lines) no such correlation was found (Allalunis-Turner et al, 1995; Nicolas et al, 1996; Lu et al, 1998). Up to now, there is only one human glioma cell line known in which radiosensitivity clearly correlates with a DNA-PKcs transcriptional defect (Lees-Miller et al, 1995).

This discrepancy between rodent and human data was the basis of the present study, in which we wanted to investigate whether in normal human fibroblasts the cellular radiosensitivity and the extent of double-strand break repair also depended on the DNA-PK complex. The study was performed with 11 normal human fibroblast lines showing a clear difference in dsb repair capacity that correlated with the cellular radiosensitivity (Dikomey et al, 1999). The expression of the *Ku70* and the *Ku80* gene was determined by Northern blot analysis and the activity of the DNA-PK complex was detected by the pulldown assay (Finnie et al, 1995).

*Received 20 April 1998*
*Revised 15 July 1998*
*Accepted 16 July 1998*

*Correspondence to:* E Dikomey

### MATERIALS AND METHODS

### Cell culture

The human skin fibroblast lines used were originally established from skin biopsies of women who had received post-mastectomy radiotherapy 10–12 years ago (Johansen et al, 1994, 1996). The biopsies were taken from non-irradiated areas. Fibroblasts were grown as a monolayer in Amniomax C100 medium containing 15% supplement and fetal calf serum (7.5% each). CHO-K1 and xrs5 cells were grown in minimal essential medium (MEM), α-modified, containing 5% fetal calf serum (FCS). All cell cultures were incubated at 37°C, 8% carbon dioxide and 100% humidification. All experiments were performed with plateau phase cultures, the human fibroblasts being in passages 6–8.

### Northern blot hybridization

Total RNA was extracted according to the Fast-Prep System (Dianova, Hamburg, Germany). Total RNA (5 μg) was run in a 1% denaturing agarose gel. Subsequently, RNA was transferred to a nylon membrane (Hybond N+, Amersham, Braunschweig, Germany) and was fixed by UVC cross-linking.

Nylon membranes were prehybridized for 7 h in hybridization buffer containing 50% deionized formamide, 20% 5× buffer [250 mM tris-HCl pH 7.5, 1% bovine serum albumin (BSA) fraction 5, 1% polyvinylpyrrolidone, 0.5% sodium pyrophosphate, 5% sodium dodecyl sulphate (SDS), 1% Ficoll 400], 20% dextransulphate (50%), 1 M sodium chloride, 0.1 mg $ml^{-1}$ calf thymus DNA (ultrasound degraded, GibcoBRL, Eggenstein, Germany) at the appropriate hybridization temperature. After addition of the heat-denatured specific DIG-labelled probe, hybridization was carried out overnight at the appropriate temperature (β-actin probe, 42°C; Ku70 probe, 48°C; Ku80 probe, 42°C). After stringency washes (2 × 20 min, 2 × SSC + 0.1% SDS, room temperature; 2 × 15 min, 0.5 × SSC + 0.1% SDS, appropriate temperature, i.e. β-actin probe, 47°C; Ku70 probe, 66°C; and Ku80 probe, 52°C), the hybridization signal was detected via chemiluminescence by using CDP-Star (Boehringer, Mannheim, Germany). Light emission was detected either by exposure of a sensitive film (Hyperfilm ECL, Amersham) or by direct recording and subsequent analysis (Molecular Light Imager – Night OWL; software, winlight; Berthold, Isernhagen, Germany).

Nylon membranes were stripped with Northern probe-stripping solution (50% formamide, 50 mM tris-HCl pH 8, 1% SDS) according to the Boehringer manual (Boehringer).

For the preparation of the specific DNA probes, XL1 Blue bacteria [genotype recA1, endA1, gyrA96, thi-1, hsdR17, supE44, relA1, lac, (F′traD36, proAB, lacIPZΔM15, Tn10(tet))] were used, which were transformed with pGem-plasmid containing the human cDNA of either the *Ku70* or the *Ku80* gene (p70-4z or p80-4z) and which were kindly provided by Dr H Feldmann, Munich, Germany. Bacteria were grown in LB-medium containing 50 μg $ml^{-1}$ ampicillin. Plasmids were isolated according to the Plasmid Maxi Kit (Qiagen, Hilden, Germany) and were cut with the appropriate restriction enzymes (p70-4z, *Eco*R1, *Pst*1; p80-4z, *Pst*1, *Sma*1; Pharmacia Biotech, Freiburg, Germany). Reaction products were separated in a 1% non-denaturing agarose gel. The resulting DNA fragments representing the specific DNA probes (Ku70, ~1000 bp; Ku80, ~400 bp) were isolated according to the QIAquick Kit (Qiagen). These two probes were DIG-labelled by the random primed labelling method (Boehringer). As specific β-actin probe, a 40-base single-stranded synthetic oligonucleotide (62.5% GC content, Oncogene Science, Dianova, Hamburg, Germany) was used, which was DIG-labelled by the 3′ end-labelling method (Boehringer).

### DNA-PK pulldown assay

Whole cell extracts were prepared according to Finnie et al (1995). Trypsinized cells were washed once with phosphate-buffered saline (140 mM Sodium chloride, 3 mM potassium chloride, 8 mM disodium hydrogen phosphate dihydrate, 1 mM potassium dihydrogen phosphate) and were precipitated. The cell pellet was resuspended in one pellet volume extraction buffer (50 mM sodium fluoride, 20 mM Hepes pH 7.6, 450 mM sodium chloride, 25% glycerol, 0.2 mM EDTA, 0.5 mM dithiothreitol, 0.5 mM phenylmethylsulphonyl fluoride, 0.5 μg $ml^{-1}$ leupeptin, 0.5 μg $ml^{-1}$ pepstatin A protease inhibitor, 1.0 mg $ml^{-1}$ soybean trypsin inhibitor, 0.5 μg $ml^{-1}$ aprotinin, 40 μg $ml^{-1}$ bestatin). The suspension was frozen in liquid nitrogen and, subsequently, was thawed at 30°C, four times each. After centrifugation at 11 000 r.p.m. (10 min, 4°C), the supernatant (i.e. the cell extract) was stored at –70°C. The protein concentration of this whole cell extract was determined by the BioRad-Microassay procedure (BioRad, Munich, Germany).

The DNA-PK activity was determined by the pulldown assay (Finnie et al, 1995). In this assay, the DNA-PK firstly was purified from the extract, and subsequently the enzyme activity was determined by its kinase reaction. For the purification step, calf thymus dsDNA-cellulose (Sigma, Deisenhofen, Germany) was swollen in buffer Z (25 mM Hepes pH 7.6, 12.5 mM magnesium chloride, 20% glycerol, 0.1% Nonidet-P40, 1 mM dithiothreitol, 50 mM potassium chloride) for 2–24 h at 4°C. After one washing step, the dsDNA-cellulose pellet was resuspended in one pellet volume of buffer Z. Twenty microlitres of this dsDNA-cellulose suspension was incubated with the indicated amount of protein of the whole cell extract for 1 h at 4°C on a rotating wheel. Thereafter, dsDNA-cellulose was washed once with 1 ml buffer Z, and the dsDNA-cellulose pellet was resuspended in 60 μl buffer Z. The enzyme reaction was carried out at 30°C for 30 min and was set up as follows: 10 μl resuspended dsDNA-cellulose, 6 μl buffer Z, 2 μl peptide (2 mM), 2 μl ATP mix; 19.5 μl dATP (2 mM, unlabelled, Amersham) with 0.5 μl [$^{32}$P]ATP (>5000 Ci $mmol^{-1}$, Amersham) mixed before usage. The peptides used represent a sequence of the human p53 protein (Lees-Miller et al, 1992); specific peptide, $NH_2$-EPPQSQE-AFADLWKK-COOH (Eurogentec, Seraing, Belgium); mutated peptide, $NH_2$-EPPLSEQAFADLLKK-COOH (friendly gift of Dr J Coco Martin, Amsterdam, The Netherlands). The enzyme reaction was stopped by adding 30 μl acetic acid (30%). Twenty microlitres of each sample was spotted onto a piece of phosphocellulose paper (P81, Whatman, Göttingen, Germany). These P81 pieces were washed four times with acetic acid (15%), and subsequently the decay of incorporated $^{32}$P was counted by liquid scintillation counting (Tri-Carb, Packard, Frankfurt a.M, Germany).

### RESULTS

### Gene expression of *Ku70* and *Ku80*

The expression of the two DNA repair genes *Ku70* and *Ku80* was examined in 11 different normal human fibroblast lines. The cells were grown to confluence and the gene expression was detected via chemiluminescence by using DIG-labelled specific DNA probes. As demonstrated in Figure 1, each of the tested cell lines possesses a *Ku70* signal of about 2.2 kb and two variants of *Ku80* transcripts of about 2.4 kb and 3.5 kb, respectively, which is in agreement with literature (Cai et al, 1994). As a control for gel loading, the signal intensity of *β-actin* was used (Figure 1C).

 *© Cancer Research Campaign 1999*

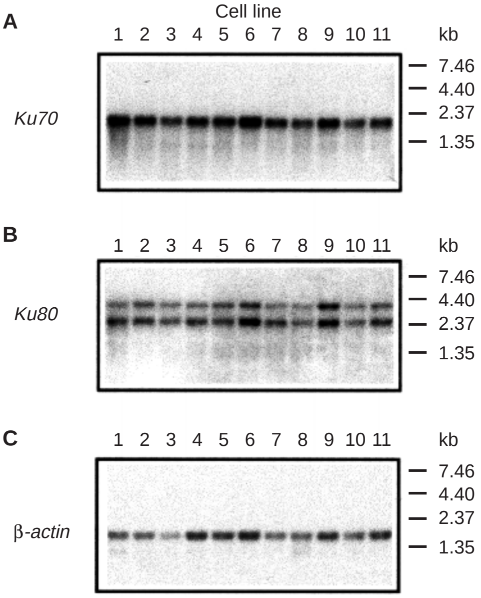

**Figure 1** Gene expression of *Ku70*, *Ku80* and *β-actin* of 11 different normal human fibroblast lines. Total RNA (5 µg) was analysed by Northern blot, hybridization with the appropriate DIG-labelled DNA probe and subsequent chemiluminescence detection via exposure of a light-sensitive film

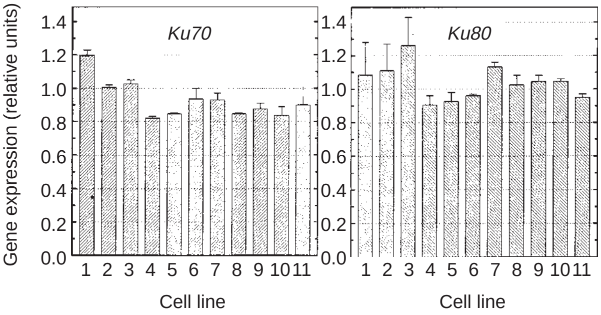

**Figure 2** Relative gene expression of *Ku70* and *Ku80* of 11 different normal human fibroblast lines. Data were obtained from blots like that one shown in Figure 1, however the hybridization signals were determined directly via the light emission recorded by a highly sensitive camera. The data were normalized to the *β-actin* signal to correct for gel loading. The *Ku80* value represents the sum of both transcripts. For better comparison, all values were normalized to the mean value. Error bars represent s.e.m. of three independent RNA preparations

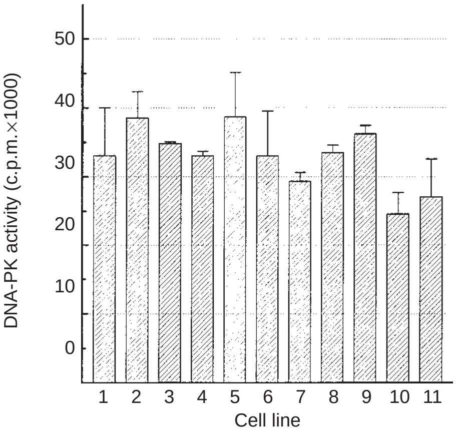

**Figure 3** Activity of DNA-PK determined for 11 normal human fibroblast lines. Protein (150 µg) of the appropriate whole cell extract was used. All data were corrected for the background, i.e. c.p.m. in the absence of peptide. The error bars represent s.e.m. of two independent experiments, with triplicate determination

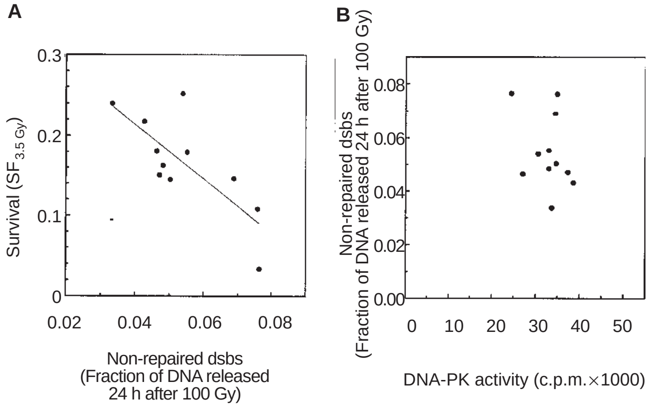

**Figure 4** Relation between cellular survival, double-strand break repair capacity and DNA-PK activity in 11 normal human fibroblast lines. (**A**) Correlation between cell survival and dsb repair capacity. Cell survival after 3.5 Gy was determined for plateau phase cells by colony-forming ability, with delayed plating (14 h) after irradiation; the repair capacity was expressed by the number of non-repaired dsbs detected by constant field gel electrophoresis as a fraction of DNA released from the plug 24 h after irradiation with 100 Gy; data were taken from Dikomey et al (1999). (**B**) Lack of correlation between dsb repair capacity and DNA-PK activity. The number of non-repaired dsbs were determined as described above; data of DNA-PK activity were taken from Figure 3

Figure 2 shows the expression of *Ku70* and *Ku80* as corrected for gel loading using the *β-actin* signal. The *Ku80* value represents the sum of both transcripts. For a better comparison, all values were normalized to the mean value. On average, the deviation from the mean was very small, with ±9% for *Ku70* and ±8% for *Ku80*, respectively, showing that the expression of these genes is fairly constant for the 11 cell lines. The small differences observed were considered to result from variations in the preparation of the total RNA rather than from biological differences in the level of gene expression because the ranking of gene expression varied between experiments with different RNA preparations (as indicated by the error bars).

### DNA-PK activity

The activity of the DNA-PK (which represents a serine/ threonine kinase) was determined by the pulldown assay (Finnie et al, 1995). The specificity of this assay was tested by determination of the $^{32}P$ transfer onto a specific peptide compared with the transfer onto a mutated peptide, which lacks a DNA-PK-specific phosphorylation site, or the transfer in the absence of peptide for background evaluation. In the presence of the specific peptide, there was a clear kinase activity. In contrast, using the mutated peptide, the activity detected was similar to the background level (data not shown). These findings clearly demonstrated that the activity detected results from the DNA-PK complex and not from other kinases.

Figure 3 represents the DNA-PK activity measured for the 11 human fibroblast lines after application of 150 µg protein of the appropriate whole cell extract using the specific peptide. On average, the variation was very small with a mean deviation of ±11%. The variations in the DNA-PK activity observed are in the range of the standard errors, showing that these variations mainly result from experimental errors but not from biologically different levels of DNA-PK activity.

© Cancer Research Campaign 1999

### Relation between DNA double-strand break repair capacity and DNA-PK activity

The human fibroblast lines used in the present study were previously shown to differ in their cellular radiosensitivity, with the surviving fraction measured for a dose of 3.5 Gy, $SF_{3.5\ Gy}$, ranging from 0.03 to 0.28. These variations in radiosensitivity correlate well with the double-strand break repair capacity, as demonstrated by the number of non-repaired double-strand breaks detected 24 h after irradiation with 100 Gy (Figure 4A). However, as demonstrated in Figure 4B, the double-strand break repair capacity of these fibroblast lines does not correlate with the DNA-PK activity. Cell lines with distinct differences in the number of non-repaired double-strand breaks did not show a variation in the DNA-PK activity.

### DISCUSSION

The knowledge of proteins defining the cellular radiosensitivity is of profound interest in understanding the clinical phenomenon of different, individual radiosensitivity. In the present study, it was tested for the first time whether the cellular radiosensitivity and double-strand break repair capacity of normal human fibroblasts is determined by the activity of the DNA-dependent protein kinase. This complex is known to participate to a non-homologous recombination pathway, which is the dominant repair mechanism active in mammalian cells (Jackson and Jeggo, 1995).

The experiments were carried out with confluent cultures mainly consisting of $G_1$ cells (>95%) to avoid cell cycle-dependent alterations because the DNA-PK activity is known to vary with the cell cycle with a maximum in $G_1$ and a minimum in the S-phase (Lee et al, 1997). In these 11 cultures, both the level of gene expression tested for *Ku70* and *Ku80* and the activity of the DNA-PK were found to show only a fairly small variation (Figure 2 and Figure 3). On average, the deviation from the mean was less than 9% for the gene expression and 11% for the DNA-PK activity.

The 11 fibroblast lines used are known to exhibit a typical spectrum of normal radiosensitivity with $SF_{3.5\ Gy}$ ranging from 0.03 to 0.28 (Dikomey et al, 1999). It was demonstrated here that the variation in cellular radiosensitivity found for human fibroblasts cannot be attributed to a variation in DNA-PK activity. Similar results were previously obtained for ten human tumour cell lines (Allalunis-Turner, 1995) and for a sensitive non-tumour cell line (Lu et al, 1998). Only for the human glioma line MO59J could the increase in cellular radiosensitivity be associated with a defect in the DNA-PK complex (Lees-Miller et al, 1995).

It was also shown here that in human fibroblasts the DNA-PK activity did not correlate with double-strand break repair capacity (Figure 4B), although these fibroblasts were previously observed to show a broad range in repair capacity which was found to correlate with the respective cellular radiosensitivity (Figure 4A, Dikomey et al, 1999). This result illustrates that in human fibroblasts the variation in double-strand break repair capacity cannot result from differences in the DNA-PK activity. These results are in contrast to data obtained for rodent cell lines of the complementation groups 5–7, for which an increase in radiosensitivity could be ascribed to a decrease in the DNA-PK activity (Weaver, 1995). However, these cell lines cannot be used as a general model of the impact of the DNA-PK complex because the reduction of DNA-PK activity was always caused by a genetic defect and not by a variation in the transcription or translation level. Probably, an activity of the DNA-PK other than the kinase activity is relevant for double-strand break repair, but this activity then has to be independent from the kinase activity.

In this context, it is also important to note that in normal human fibroblasts the activity of DNA-PK was about 70 times higher than in normal rodent cells (data not shown; Anderson and Lees-Miller, 1992). This difference, however, did not result in an enhanced repair capacity of human cells (Dikomey et al, 1998). This observation indicates that the DNA-PK complex is not rate limiting for dsb repair. Probably, other enzymes that are less expressed compared with the DNA-PK are rate limiting and, therefore, more relevant for double-strand break repair. From the eight genes identified so far to affect the cellular radiosensitivity of mammalian cells (*XRCC1–XRCC8*), the three genes *XRCC5–XRCC7* can be excluded from determining the double-strand break repair capacity of human fibroblast lines because of the data shown here. Also, the *XRCC1* and the *XRCC3* genes can be excluded because the products of these genes are known to participate in single- but not in double-strand break repair (Zdzienicka, 1995). In this respect, the *XRCC8* gene is also not relevant, because cells mutated in this gene show normal single-strand break and double-strand break repair (Zdzienicka, 1995). The *XRCC2* gene product can also be rejected because a defect in this gene was found only to affect the fidelity but not the capacity of double-strand break repair (Zdzienicka, 1995). Probably, the *XRCC4* gene is of relevance. It was recently shown that the corresponding gene product is a stimulating factor of DNA ligase IV, acting downstream of the Ku70/Ku80 proteins and thereby representing part of the DNA-PK pathway (Critchlow et al, 1997; Grawunder et al, 1997; Wilson et al, 1997). Also the *ATM* gene might be of importance because a defect in this gene was found to result in a reduced double-strand break repair capacity (Dikomey et al, 1998; Blöcher et al, 1991).

It should be kept in mind that cell lines defective in the DNA-PK pathway are still able to repair more than 50% of the double-strand breaks induced (Kemp et al, 1984; Hendrickson et al, 1991). These double-strand breaks are repaired either by another non-homologous recombination pathway or even by homologous recombination, and it remains to be shown whether cellular radiosensitivity is mainly determined by these mechanisms.

In conclusion, the data presented demonstrate that in normal human fibroblast lines double-strand break repair capacity and cellular radiosensitivity do not correlate with the DNA-PK activity. Obviously, in this respect other proteins or pathways are more relevant.

### ACKNOWLEDGEMENTS

The authors thank Dr Heidi Feldmann (Institute of Biochemistry, University of Munich) for the kindly gift of the Ku70/Ku80 transfected bacteria XL1 Blue and Dr Jose Coco Martin (The Netherlands Cancer Institute, Amsterdam) for the mutated peptide. We also thank Professor Dr Armin Hildebrandt (UFT, University of Bremen) for the feasibility to use the highly sensitive camera system and especially Andreas Marquardt and Manfred Pisula for their practical help. In addition, we are grateful to Kerstin Borgmann, Dr Jochen Dahm-Daphi and Dr Ingo Brammer for valuable discussions.

This work was supported by the Deutsche Forschungsgemeinschaft, grant no. Di 457/2–1 (ED), and by the Danish Cancer Society, grant no. 9510054 (JO), and by the BIOMED project no. PL950638.

 © Cancer Research Campaign 1999

### ABBREVIATIONS

DIG, Digoxigenin; DNA-PK, DNA-dependent protein kinase; dsb, double-strand break; dsDNA, double-stranded DNA; SF, surviving fraction; XRCC, X-ray cross complementing.

### REFERENCES

Allalunis-Turner MJ, Lintott LG, Barron GM, Day RS and Lees-Miller S (1995) Lack of correlation between DNA-dependent protein kinase activity and tumor cell radiosensitivity. *Cancer Res* **55**: 5200–5202

Anderson CW and Lees-Miller SP (1992) The nuclear serine/threonine protein kinase DNA-PK. *Crit Rev Eukaryot Gene Express* **2**: 283–314

Bentzen SM and Overgaard J (1994) Patient-to-patient variability in the expression of radiation-induced normal tissue injury. *Semin Radiat Oncol* **4**: 68–80

Blöcher D, Sigut D and Hannan MA (1991) Fibroblasts from ataxia telangiectasia (AT) and AT heterozygotes show an enhanced level of residual DNA double-strand breaks after low dose-rate γ-irradiation as assayed by pulsed field gel electrophoresis. *Int J Radiat Biol* **60**: 791–802

Burnet NG, Wurm R and Peacock JH (1996) Low dose-rate fibroblast radiosensitivity and the prediction of patient response to radiotherapy. *Int J Radiat Biol* **70**: 289–300

Cai QQ, Plet A, Imbert J, Lafage-Pochitaloff M, Cerdan C and Blanchard JM (1994) Chromosomal location and expression of the genes coding for Ku p70 and p80 in human cell lines and normal tissues. *Cytogenet Cell Genet* **65**: 221–227

Cornforth MN and Bedford JS (1987) A quantitative comparison of potentially lethal damage repair and the rejoining of interphase chromosome breaks in low passage normal human fibroblasts. *Radiat Res* **111**: 385–405

Critchlow SE, Bowater RP and Jackson SP (1997) Mammalian DNA double-strand break repair protein XRCC4 interacts with DNA ligase IV. *Curr Biol* **7**: 588–598

Dikomey E, Dahm-Daphi J, Brammer I, Martensen R and Kaina B (1998) Correlation between cellular radiosensitivity and non-repaired double-strand breaks studied in nine mammalian cell lines. *Int J Radiat Biol* **73**: 269–278

Dikomey E, Brammer I, Johansen J, Bentzen SM and Overgaard J (1999) Residual DNA double-strand breaks measured in confluent skin fibroblasts correlate with cell killing but not with the risk of fibrosis of the patients. *Int J Radiat Oncol Biol Phys* (submitted)

Di Leonardo A, Linke SP, Clarkin K and Wahl GM (1994) DNA damage triggers a prolonged p53-dependent G1 arrest and long-term induction of Cip1 in normal human fibroblasts. *Genes Dev* **8**: 2540–2551

Finnie NJ, Gottlieb TM, Blunt T, Jeggo PA and Jackson SP (1995) DNA-dependent protein kinase activity is absent in xrs-6 cells: implications for site-specific recombination and DNA double-strand break repair. *Proc Natl Acad Sci USA* **92**: 320–324

Geara FB, Peters LJ, Kian Ang K, Wike JL and Brock WA (1993) Prospective comparison of in vitro normal cell radiosensitivity and normal tissue reactions in radiotherapy patients. *Int J Radiat Oncol Biol Phys* **27**: 1173–1179

Grawunder U, Wilm M, Wu X, Kulesza P, Wilson TE, Mann M and Lieber MR (1997) Activity of DNA ligase IV stimulated by complex formation with XRCC4 protein in mammalian cells. *Nature* **388**: 492–495

Hendrickson EA, Qin XQ, Bump EA, Schatz DG, Oettinger M and Weaver DT (1991) A link between double-strand break-related repair and V(D)J recombination: the scid mutation. *Proc Natl Acad Sci USA* **88**: 4061–4065

Jackson SP and Jeggo PA (1995) DNA double-strand break repair and V(D)J recombination: involvement of DNA-PK. *Trends Biol Sci* **20**: 412–415

Johansen J, Bentzen SM, Overgaard J and Overgaard M (1994) Evidence for a positive correlation between in vitro radiosensitivity of normal human skin fibroblasts and the occurrence of subcutaneous fibrosis after radiotherapy. *Int J Radiat Biol* **66**: 407–412

Johansen J, Bentzen SM, Overgaard J and Overgaard M (1996) Relationship between the in vitro radiosensitivity of skin fibroblasts and the expression of subcutaneous fibrosis, telangiectasia, and skin erythema after radiotherapy. *Radiother Oncol* **40**: 101–109

Kemp LM, Sedgwick SG and Jeggo PA (1984) X-ray sensitive mutants of Chinese hamster ovary cells defective in double-strand break rejoining. *Mutat Res* **132**: 189–196

Lee SE, Mitchell RA, Cheng A and Hendrickson EA (1997) Evidence for DNA-PK-dependent and -independent DNA double-strand break repair pathways in mammalian cells as a function of the cell cycle. *Mol Cell Biol* **17**: 1425–1433

Lees-Miller SP, Sakaguchi K, Ullrich SJ, Appella E and Anderson CW (1992) Human DNA-activated protein kinase phosphorylates serines 15 and 37 in the amino-terminal transactivation domain of human p53. *Mol Cell Biol* **12**: 5041–5049

Lees-Miller SP, Godbout R, Chan DW, Weinfeld M, Day RS, Barron GM and Allalunis-Turner J (1995) Absence of p350 subunit of DNA-activated protein kinase from a radiosensitive human cell line. *Science* **267**: 1183–1185

Lu H, Song Q, Arlett C and Lavin MF (1998) The radiosensitive cell line 180BR is not defective in the major DNA damage-sensing proteins. *Cancer Res* **58**: 84–88

Myung K, He DM, Lee SE and Hendrickson EA (1997) KARP-1: a novel leucine zipper protein expressed from the Ku86 autoantigen locus is implicated in the control of DNA-dependent protein kinase activity. *EMBOJ* **16**: 3172–3184

Nicolas N, Finnie NJ, Cavazzana-Calvo M, Papadopoulo D, Le Deist F, Fisher A, Jackson SP and de Villartay JP (1996) Lack of detectable defect in DNA double-strand break repair and DNA-dependent protein kinase activity in radiosensitive human severe combined immunodeficiency fibroblasts. *Eur J Immunol* **26**: 1118–1122

Ramsay J and Birrell G (1995) Normal tissue radiosensitivity in breast cancer patients. *Int J Radiat Oncol Biol Phys* **32**: 339–344

Turesson I, Nyman J, Holmberg E and Oden A (1996) Prognostic factors for acute and late skin reactions in radiotherapy patients. *Int J Radiat Oncol Biol Phys* **36**: 1065–1075

Weaver DT (1995) What to do at an end: DNA double-strand-break repair. *Trends Genet* **11**: 388–392

Wilson TE, Grawunder U and Lieber MR (1997) Yeast DNA ligase IV mediates non-homologous DNA end joining. *Nature* **388**: 495–498

Zdzienicka MZ (1995) Mammalian mutants defective in the response to ionizing radiation-induced DNA damage. *Mutat Res* **336**: 203–213

*© Cancer Research Campaign 1999* 
